# Editorial

**DOI:** 10.1007/s12064-024-00430-7

**Published:** 2024-11-20

**Authors:** Jürgen Jost

**Affiliations:** Max Planck Institute for Mathematics in the Sciences, Leipzig, Germany

The journal *Theory in Biosciences* has a long and distinguished history. It was founded in 1881 as *Biologisches Centralblatt* by Isidor Rosenthal, Professor of Physiology in Erlangen, a former student of Emil Du Bois-Reymond. He was supported by the Professor of Botany Maximilian Reeß and the Professor of Zoology Emil Selenka. The new journal published not only original research articles, but also essays, surveys, and book reviews. In 1915, Rosenthal passed away, and in 1917 Ernst Weinland, who had obtained Rosenthal’s chair in 1913 and was the main editor from 1917 to 1920, changed the spelling to *Biologisches Zentralblatt*. After several transitions, its publisher was Gustav Fischer in Jena when Olaf Breidbach, since 1995 Professor for the History of the Sciences in Jena, became its editor-in-chief. He changed the scope of the journal and its title to *Theory in Biosciences—Theorie in den Biowissenschaften* and edited it together with Michael Weingarten. The local publisher Gustav Fischer was then swallowed by Elsevier, and since 2004, the journal was thus published by Elsevier. In that year, Peter Stadler, Professor of Bioinformatics at Leipzig, and myself joined the editorial board, to strengthen the rapidly developing fields of computational, mathematical, and theoretical biology (see the editorial Breidbach et al. 2004). But since Elsevier found that the journal did not make enough money, in 2007, we received a rather rude letter (for instance, misspelling even two out of our three names) that they wanted to discontinue the journal. Since I had already good relations with Springer, we could convince that publisher to continue the journal (see the editorial Breidbach et al. 2007). I am grateful for the support by competent and dedicated staff.

*Theory in Biosciences* has since found its place as a journal in theoretical, mathematical, and computational biology and on the history of biology. When Olaf Breidbach became seriously ill in 2013 and sadly passed away in 2014, I took over the role and the duty of the managing editor, and at the end of this year, I shall hand it over to Peter Stadler.

As indicated, Olaf Breidbach had transformed the *Biologisches Zentralblatt* into *Theory in Biosciences*, thereby changing and expanding the scope of the journal. He has been commemorated in this journal by an obituary (Jost 2014) and two special issues (Jost 2016, 2017) from which the reader can gain a good impression of the scope of the journal. Olaf Breidbach had also initiated several special issues. These and more recent such issues critically reflect fundamental theoretical issues (e.g. Gutmann et al. 2000; Daniels et al. 2021), cover topics in contemporary biology experiencing rapid development (e.g. Breidbach 2009; Boi and Lobo 2022), or provide a perspective on historical developments (e.g. Hoßfeld et al. 2003; Kutschera and Hoßfeld 2013; Kutschera et al. 2019). Such special issues can thus identify and set important themes for a thorough theoretical discussion. The preceding selection already indicates the wide range of topics covered in our journal.

Biology has profoundly changed since the beginning of this journal in 1881, and its scientific standing as well as its impact for our lives and our society has dramatically increased. In the late nineteenth century, obviously the rise of the theory of evolution of Darwin and Wallace and its German advocat Haeckel was the most important development, at least in retrospect. Classical morphology, as the science mediating between structure and function, was challenged by evolutionary thinking and turned into a tool for the reconstruction of ancestral relations. Developmental biology, started earlier by von Baer, still had to be integrated into an evolutionary perspective, attempted by Haeckel. The concept of a gene had been conceived by Mendel, but was not yet known in the community. (For the history of the rediscovery, an important paper (Simunek et al. 2012) appeared in our journal.) The integration of the theory of evolution and genetics later lead to the so-called Neodarwinian synthesis. This also triggered an important development in mathematical biology, population genetics, with the foundational contributions of Fisher, Wright, and Kimura. And this was later supplemented by evolutionary game theory. But biology moved on, and became dominated by biochemistry and molecular biology. Again, this also triggered theoretical and mathematical developments, and in particular, the new fields of bioinformatics and computational biology emerged. For instance, evolutionary trees are now usually reconstructed from genetic sequence data instead of from fossils. These days, we see another profound shift in biology, from the analysis of individual molecular structures and the understanding of particular processes and pathways to the generation of data covering entire genomes or even cells, and from bulk data to those involving individual cells. This creates new challenges which are addressed by methods from machine learning and tools from AI, often with spectacular results like those achieved by AlphaFold for the computation of protein structures from sequence data.

Also the neurosciences are making important advances, at scales ranging from the molecular level to entire brains, and again the need for computational tools, mathematical methods, and theoretical concepts is apparent.

I hope that *Theory in Biosciences* will continue to contribute to all these developments and at the same time provide a deep historical perspective.

I would like to thank the authors of *Theory in Biosciences* for many innovative and thought provoking articles and the many editors and editorial board members that have helped to run the journal over its long and distinguished history, and I wish the journal all the best for its future in the hands of Peter Stadler and Sonja Prohaska.
